# Dissecting Microscopic Colitis Immunopathophysiology: Insights From Basic Research

**DOI:** 10.1002/ueg2.70024

**Published:** 2025-05-03

**Authors:** Andreas Münch, Celia Escudero‐Hernández

**Affiliations:** ^1^ Department of Gastroenterology and Hepatology Linköping University Linköping Sweden; ^2^ Department of Health, Medicine, and Caring Sciences Linköping University Linköping Sweden; ^3^ Institute of Clinical Molecular Biology (IKMB) Christian‐Albrechts‐University and University Hospital Schleswig‐Holstein Kiel Germany

**Keywords:** collagenous colitis, *HLA*genes, immunopathophysiology, inflammatory bowel disease (IBD), lymphocytic colitis, malabsorption, microscopic colitis, multiomic approaches, precision medicine

## Abstract

Microscopic colitis is an inflammatory bowel disease (IBD) comprising two clinically undiscernible entities: collagenous colitis and lymphocytic colitis. Collagenous colitis associates with *HLA* genes and displays a Th1/Tc1–Th17/Tc17 profile with pericryptal myofibroblast activity, water malabsorption and secondary fluid loss due to altered osmoregulation. Conversely, lymphocytic colitis lacks genetic associations and displays a Th1/Th2 profile and paracellular/transcellular permeability. Lymphocytic colitis subclassifies into channelopathic lymphocytic colitis due to unique alteration of ion and organic acid transport that could result from drug exposure, and inflammatory lymphocytic colitis due to the involvement of moderate immune responses compared to collagenous colitis. As microscopic colitis mucosa remains intact and immune cells seem to stay inactive, microscopic colitis is an ideal model to explore early stages of IBD if collagenous colitis and lymphocytic colitis are studied as distinct entities. Exploiting multiomic approaches and established biobanks will ensure validation of microscopic colitis patient stratification, and deepening into pathomechanisms which could enable precision medicine.

## Graphical abstract

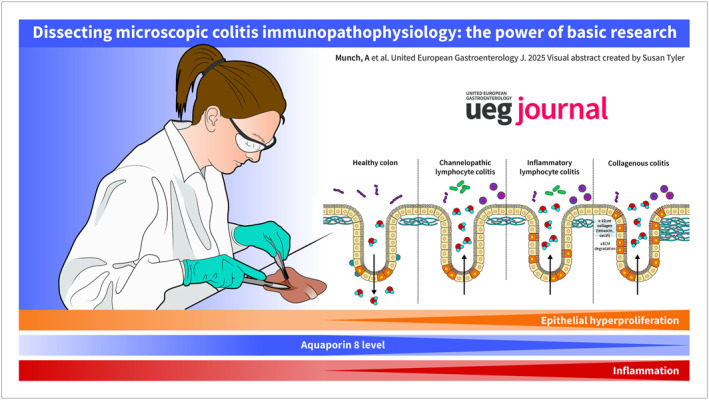

**Abbreviations**
CC: collagenous colitis
CD: Crohn's disease
IBD: inflammatory bowel disease
LC: lymphocytic colitis
UC: ulcerative colitis

## 1 Introduction

**FIGURE 1:**
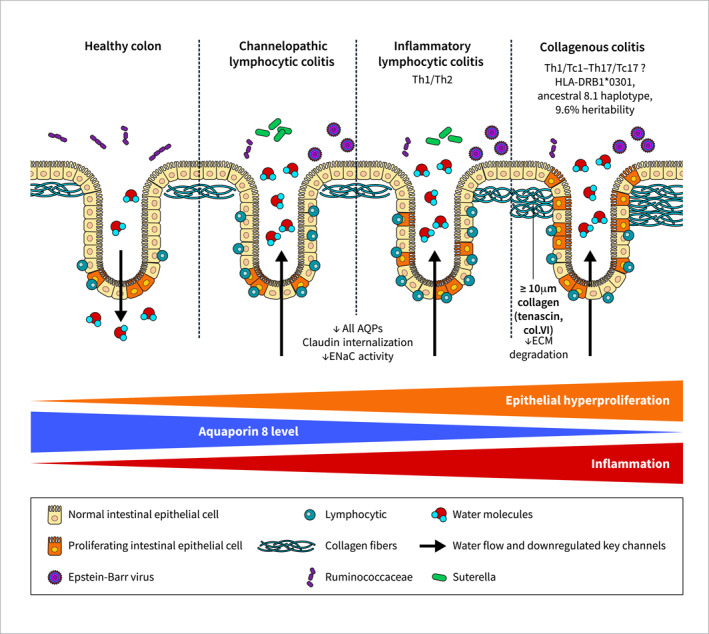
Distinct characteristics between microscopic colitis subtypes. Microscopic colitis subtypes display an increasing gradient of inflammation and intestinal crypt hyperproliferation, ranging from channelopathic lymphocytic colitis to inflammatory lymphocytic colitis and collagenous colitis. Similarly, a decreasing gradient of water channel aquaporin (AQP) 8 expression occurs. Collagenous colitis is genetically associated with HLA genes and presumably displays a Th1/Tc1–Th17/Tc17 immune response and a thickened subepithelial collagenous band (> 10 μm) resulting from defects in the degradation of extracellular matrix (ECM) proteins. On the other hand, lymphocytic colitis inflammation qualifies as Th1/Th2 and lacks genetic associations. Expression of major ion channels accounting for water malabsorption (AQP8), osmotic fluid loss (*SLC9A3*/NHE3, *SLC26A3*/DRA), and epithelial permeability (claudins) differ between microscopic colitis subtypes. In addition, the butyrate‐producing Ruminococcaceae bacterial family is underrepresented in microscopic colitis because of watery diarrhoea washing, while Epstein‐Barr virus (and Suterella in lymphocytic colitis) is overrepresented.

Microscopic colitis (MC) is an inflammatory bowel disease (IBD) and a common cause of chronic, watery and non‐bloody diarrhoea. Due to the increasing awareness that these patients should be referred to colonoscopy with biopsies, current incidence is estimated to reach that of classic IBD Crohn's disease (CD) and ulcerative colitis (UC) [1]. Since MC lacks macroscopic mucosal damage, diagnose relies in histology and splits MC into collagenous colitis (CC) due to the thickened subepithelial collagenous band (> 10 μm), and lymphocytic colitis (LC) due to its significant intraepithelial lymphocytosis (> 20 lymphocytes per 100 epithelial cells, Figure 1) [2]. However, histological findings do not correlate with disease activity, that is, stool frequency [3]. From a clinical point of view, CC and LC are not distinguishable and are thus treated in the same way, with the corticosteroid budesonide being the drug of choice [2]. While budesonide is an effective first‐line treatment, up to 80% patients relapse when it is discontinued and up to 7% are refractory to budesonide [1]. With the new era of advanced therapies, JAK inhibitors seem a promising alternative [4]. Still, a better understanding of MC immunopathophysiology is imperative to comprehend the molecular basis of MC to accurately target disease activity. Basic research in MC is in its infancy [5], yet it has identified key distinctive features of each MC subtype (summarised in Figure 1 and Table 1). With this review, we aim at summarising the existing literature to explain the immunopathophysiology of CC and LC—provided that these entities were considered separately, and excluded single case reports –, to identify consistent similarities and differences between these close, yet different entities.

**TABLE 1:** Distinct characteristics between microscopic colitis subtypes.

Feature	Lymphocytic colitis	Collagenous colitis
Histopathologic hallmark	> 20 lymphocytes/100 epithelial cells	> 10 μm subepithelial collagenous band due to decreased ECM degradation (tenascin and collagen type VI accumulation)
Patient stratification	Channelopathic LC, inflammatory LC	—
Genetic associations	—	HLA‐DRB1*03:01, ancestral 8.1 haplotype
Heritability	—	9.6%
Diarrhoeal mechanisms	Paracellular and transcellular permeability (↓AQPs, ↓claudins, ↓ENaCγ activity), moderate crypt hyperproliferation (all are more prominent in inflammatory LC)	Water malabsorption (↓AQP8), osmotic fluid loss (↓SLC9A3/NHE3, ↓SLC26A3/DRA), crypt hyperproliferation
Immune response	Absent (channelopathic LC), minor (inflammatory LC)	Moderate (compared to CD and UC)
Immunological profile	Th1/Th2	Th1/Tc1‐Th17/Tc17?
Possible immune brakes	Expression of Th2 transcription factor GATA3	Decreased expression of T‐cell activation marker CD69
Shared genetic risk	—	Coeliac disease, CD, UC
Clinical associated disorders	Coeliac disease	Coeliac disease, CD, UC, autoimmune thyroid disease
Promotion to classic IBD	↑IFN‐γ, ↑GATA‐3	↑TNF‐α, ↑T‐bet, ↑GATA‐3
Microbiota^a^	↓Ruminococcaceae, ↑Sutterella, ↑Epstein‐Barr virus	↓Ruminococcaceae, ↑Epstein‐Barr virus

Abbreviations: AQP, aquaporin; CD, Crohn's disease: DRA, downregulated in adenoma; ECM, extracellular matrix; GATA, GATA Binding Protein; IBD, inflammatory bowel disease; IFN, interferon; NHE, sodium‐hydrogen exchanger; SLC, solute carrier; T‐bet, T‐box expressed in T cells; Tc, T cytotoxic cell; Th, T helper cell; TNF, tumour necrosis factor; UC, ulcerative colitis.

^a^ Microbial associations that have been identified for each microscopic colitis subtype separately.

## 2 Genetics Catalogue Collagenous and Lymphocytic Colitis as Distinct Entities and Transcriptomics Splits Lymphocytic Colitis Into Two Subgroups

Explorative studies on genetical and transcriptional levels are challenging the term of MC as an umbrella definition. The last genome‐wide association study (GWAS) meta‐analysis, which included 1498 CC and 373 LC patients, highlighted human leucocyte antigen *HLA* genes as predisposing factors for CC but not LC [6]. Among these, HLA‐DRB1∗03:01 and the ancestral 8.1 haplotype dominate and imply bacterial infections [7]. The strongest association was found with HLA‐DRB1*0301 arginine 74 and asparagine 77, two amino acids located on the peptide binding groove of the HLA‐DR molecule able to determine peptide binding affinity and avidity [6, 8]. Interestingly, these residues have also been highlighted in rheumatoid arthritis with preferential binding for collagen II, and with fibrotic‐dominant primary biliary cholangitis [9, 10, 11]. In comparison with other inflammatory intestinal conditions, only CC shared genetic risk alleles with coeliac disease and CD, while sharing the closest genetic architecture with UC [12]. Based on the single‐nucleotide polymorphisms, CC heritability was estimated at 9.6%, similar to other gastrointestinal disorders such as 5.8% for irritable bowel syndrome, 9.8%–17.3% for colonic polyps or 17.8% for diverticular disease [6].

Transcriptomics of colonic mucosa confirmed the activation of innate and adaptive immune responses against bacteria in CC. These could be mediated by increased HLA class II *HLA‐DMA* expression in the intestinal epithelial apical side, increased NF‐kB activation and increased infiltration of CD1a^+^ antigen presenting cells (oppositely to HLA class I molecules such as CD1d that dominate viral responses) [13, 14, 15]. Most of this response is abolished when CC patients respond to budesonide treatment; however, expression of genes such as proinflammatory *DUOX2*, *PLA2G2A* and *CXCL9* or ion transport *SLC9A3* (coding for the sodium exchanger NHE3) is not restored to normal levels [13]. On the other hand, LC immune response is dampened compared with CC at both humoural (Ig‐related gene expression) and cellular (cytokine and chemokine profiles) levels [16]. Furthermore, LC can be transcriptomically subdivided into channelopathic and inflammatory LC despite their same clinical presentation [16]. Since channelopathic LC displays an altered organic acid and ion transport, we hypothesised that it might be induced by drug exposure (i.e. non‐steroidal anti‐inflammatory drugs, proton pump and selective serotonin reuptake inhibitors or aspirin) [1, 16]. However, intraepithelial and lamina propria lymphocyte densities fail to correlate with clinical presentation and medications; thus, drug exposure in disease aetiology is challenged [5, 17]. On the other hand, inflammatory LC presents additional activation of the immune response, although it is more moderate compared to CC (Figure 1). Moreover, LC upregulates microRNA transcript precursors compared to CD and UC that could be exploited to develop new disease‐specific biomarkers and treatments [16]. While these data defy the existing classification of MC, confirmatory basic research using extended patient cohorts will contribute to validate this novel yet provocative subcategorization.

## 3 Collagenous Colitis Displays a Mixed Th1/Tc1–Th17/Tc17 Profile While Lymphocytic Colitis Is a Th1/Th2 Disorder

Reduced production of anti‐inflammatory interleukin (IL)‐37 has been described for both LC and CC, which could increase spontaneous chemokine expression in colonic epithelial cells [18, 19]. Indeed, upregulation of several chemokines has been reported in LC and CC (CCL2, CCL3, CCL4, CXCL8, CXCL10, CX3CL1), which could be involved in the recruitment of mixed immune cell populations including eosinophils, neutrophils, macrophages and T‐cells [20]. Histopathological examination of biopsy samples confirm this inflammatory infiltrate in the lamina propria [2]. Interestingly, eosinophils largely infiltrate CC mucosa and degranulate, providing a distinguishing faecal trace of eosinophil cationic protein and protein X (ECP and EPX) able to predict CC but not LC in patients with chronic non‐bloody diarrhoea [21, 22, 23, 24]. Moreover, CC eosinophils express transforming growth factor (TGF)‐β1, a key mediator of fibrotic processes [25, 26]. Similarly, mast cells—a myeloid cell with neuroimmune functions resident of connective tissue known for secreting histamine and heparin which have a role in allergies –, are increased in number and degranulating activity in MC [27]. Despite the detection of neutrophils in MC mucosa, neutrophil‐derived faecal calprotectin—an established marker for inflammation –, fails excluding or monitoring any MC subtype [2].

In addition to the innate response, adaptive immunity overlaps in MC. LC mucosa displays an increased presence of CD3^+^ CD8^+^ T‐cells and CD3^+^ CD4^+^ TCRγδ^+^ T‐cells, and increased expression of Th1 and CD8^+^ T cell‐associated chemokines *CXCL9*, *CXCL10*, and *CXCL11* [20, 28]. Oppositely, these cells remain unchanged in CC [28]. Importantly, CD3^+^ CD4^+^ IFN^+^ Th1 and CD3^+^ CD4^+^ IL‐17A^+^ Th17 cell numbers are decreased in both LC and CC [28]. Despite these low Th1/Th17 cell numbers, *IFNG*, *TNFA*, *IL17A*, *IL21*, *IL23* gene expression levels were found to increase in 2 MC patient cohorts. [28, 29, 30]. However, mRNA expression consistently fails to correlate protein cytokine levels, so a mixed Th1/Tc1–Th17/Tc17 immune response in MC is unclear [20, 28, 29]. Furthermore, the level of Th2 transcription factor GATA‐3 in LC mucosal T‐cells is significant, with most CD4^+^ T‐cells expressing GATA‐3, and CD8^+^ T‐cells concomitantly expressing GATA‐3 and Th1 transcription factor T‐bet. Therefore, LC shows features of a Th1/Th2 immune response that distinguishes it from CC inflammation [31].

While T regulatory cells remain unchanged in the CC mucosa, a subset of non‐suppressive FoxP3^+^ effector Th‐cells increases [32]. Also in CC, the levels of the early lymphocyte activation marker CD69 are decreased [33]. This opens the question of whether immune cells recruited to LC and CC mucosa are actually active. Thus, supported by this evidence and our transcriptomic data [13, 16], we propose that MC immune cell infiltrates could preserve limited reactivity and are already recruited as ready‐to‐react sentinels.

## 4 Autoimmunity, Gender, and the Increased Risk of Microscopic Colitis in Women

Among lymphocytes, plasma cells also infiltrate into MC mucosa, albeit scarcely compared to T‐cells [2, 30, 34]. In addition, induction of immunoglobulin‐related gene expression is detected but limited to CC [16]. Regarding autoantibodies, even though without correlation with clinical symptoms, anti‐nuclear antibodies (ANA), anti‐Saccharomyces cerevisiae antibodies (ASCA), and anti‐thyroid peroxidase (TPO) antibodies are more prevalent in CC and LC than in controls, pointing to an autoimmune aetiology [5, 35]. In agreement with this, concomitant autoimmune disorders have been reported in MC, with a higher incidence in CC [36]. Among them, the strongest association was found with coeliac disease, followed by CD and UC, and autoimmune thyroid disease [37, 38, 39]. Despite the lack of genetic association of LC with *HLA* genes, its association with coeliac disease is similar to that of CC, reaching up to 6% of cases with concomitant appearance [40, 41, 42].

Genetic analyses and epidemiological studies support the association of CC with CD and UC, suggesting an evolution of MC to/from these classic IBD forms [6, 39, 43, 44]. Indeed, Li et al. suggested that CC patients overexpressing tumour necrosis factor (TNF)‐α, the Th1 transcription factor T‐bet and Th2 transcription factor GATA‐3, and LC patients overexpressing interferon (IFN)‐γ and GATA‐3, would develop classic IBD [45]. However, large patient cohorts need to verify the course of disease progression and evolution.

As in classic IBDs, coeliac disease or other autoimmune diseases, MC shows a female preponderance [46, 47]. However, no obvious association of MC with factors influencing sex hormone levels has been reported [48]. Still, exogenous hormone use (contraceptives and menopausal hormone therapy) has been associated with an increased risk of the disease [49]. In addition, bile acids—steroid acids with hormonal actions that assist fat and oil absorption –, are malabsorbed in MC mucosa, resulting in increased bacterial uptake and increased faecal bile acid excretion [50, 51, 52]. Also, bile acid receptor farnesoid‐X‐receptor (FXR) is decreased in MC mucosa [53], which reduces epithelial barrier integrity and activates inflammasome‐dependent responses in immune cells as demonstrated by FXR knock‐out murine models [54, 55]. As a result, bile acids or intermediate compounds during bile acid synthesis have been proposed as biomarkers for bile acid malabsorption in MC to allow treatment with bile acid sequestrants [56, 57, 58].

## 5 Diarrhoeal Pathomechanisms Differ Between Collagenous and Lymphocytic Colitis

Macroscopically, MC mucosa remains intact, yet intestinal epithelial transcriptional programmes are altered [13, 16]. As mentioned above, intestinal epithelial cell (IEC)‐mediated antigen presentation could play a role in CC and induce an immune response against bacteria via HLA class II molecules [13, 14]. As such, colonic luminal nitric oxide (NO) levels, IEC inducible NO synthase (iNOS) and IEC‐derived neutrophil gelatinase‐associated lipocalin (*LCN2*/NGAL) expression are increased in LC and CC, which correlate with clinical activity [59, 60]. According to MC moderate immune response, markers of acute inflammation that increase in CD and UC mucosa remain unchanged in LC and CC (e.g. calprotectin, cytoskeletal keratin 7, M‐cell marker cathepsin E), and IEC apoptotic rate remains normal (2% compared to 5% in moderately inflamed CD and UC mucosa) [60, 61, 62, 63, 64]. Still, lysozyme upregulation in both LC and CC indicates IEC differential reprogramming: LC lysozyme is expressed in the lower parts of the crypts and subepithelial macrophages, whereas CC lysozyme is detected in colonic crypts and metaplastic Paneth cells [65]. Also, epithelial hyperproliferation is remarkable in CC colonic crypts, with more moderate hyperproliferation in inflammatory LC, and normal proliferation in channelopathic LC [13, 16]. This could lead to defects in tight‐ or adherens junctions; however, occludin, ZO‐1, claudin‐1, and JAM, or β‐catenin and E‐cadherin levels remain unchanged in LC and CC [66]. Although we and others reported downregulation of the ‘tight’ claudin 4 in LC, histological examination did not reveal changes in its localisation [16, 63, 67]. Interestingly, claudin‐5 and 8 internalise and redistribute off the tight junctions in LC, which can be mimicked by TNF‐α and IFN‐γ stimuli in HT‐29/B6 colonic IEC line [63]. Moreover, TNF‐α and IFN‐γ effects can be reversed by the glucocorticoid dexamethasone, particularly by decreasing the expression of the ‘leaky’ claudin‐2 in Caco‐2 IEC line via MAPK phosphatase‐1 (MKP‐1) [68].

A major means of increased permeability in CC relies on ion and water channels, especially downregulation of water resorption channel aquaporin (AQP) 8, the most abundant AQP in the colon [69]. This dysregulated transcellular water transport positions CC pathophysiology as a water malabsorptive disorder [69]. Water malabsorption, together with secondary fluid loss due to reduced net Na^+^/Cl^−^ absorption and chloride secretion due to altered osmoregulation via *SLC9A3*/NHE3 (Na^+^/H^+^ exchanger) and *SLA26A3*/DRA (Cl^−^/HCO_3_^‐^ exchanger), may explain the massive, rapid changes in stool frequency and consistency in CC [67, 69, 70, 71]. Interestingly, *AQP8* expression levels correlate with stool frequency and consistency, and are recovered to nearly normal levels in budesonide‐responding CC [69]. Conversely, budesonide‐refractory CC mucosa fails to restore *AQP8* gene expression and resembles UC on a transcriptional level [69]. Therefore, current therapies available for UC could benefit budesonide‐unresponsive CC patients, including not only anti‐TNF antibodies and Jak inhibitors as some clinical cohorts have demonstrated [4, 72, 73], but also leucocyte/lymphocyte trafficking blocking agents targeting α4 or β7 integrin subunits, mucosal addressin cell adhesion molecule MadCAM‐1 or sphingosine‐1‐phosphate S1P [69, 74, 75, 76, 77].

Regarding LC diarrhoeal mechanisms, these seem to differ from CC. In contrast to the singular *AQP8* downregulation and intestinal epithelial hyperproliferation observed in CC, LC densely dysregulates many AQPs and tight‐junction claudin expression in the mucosa, whereas epithelial hyperproliferation is more moderate (Figure 1). Complementarily, sodium absorption via epithelial Na^+^ channel ENaC activity is also impaired in LC due to inflammatory effector cytokines that inhibit its aldosterone‐dependent upregulation via MEK‐1/2 [78]. Thus, we suggest that an altered paracellular and transcellular ion transport occurs in LC, which distinguishes it from CC water malabsorption [13, 16]. This, together with the progressive inflammation that we noticed at transcriptional levels and ranges from absent (channelopathic LC) to minor (inflammatory LC), moderate (CC) and severe (CD and UC, Figure 1), allows us to urge for further research taking LC and CC as models to study diarrhoeal pathomechanisms and the initial stages of intestinal inflammation in a macroscopically normal mucosa.

## 6 Collagen Deposition Results From Myofibroblast Activity in Pericryptal Areas and Decreased Matrix Degradation

A distinctive feature of CC is the thickened subepithelial collagenous band. Already highlighted by Hwang et al. in 1986 and confirmed by Balázs et al. in 1988 following electron microscopy observations, pericryptal fibroblasts are less abundant but increase in size in CC and show enhanced migration and fibre‐forming activities [79, 80]. Also, the middle and upper thirds of the pericryptal fibroblast sheath are separated from the epithelium and assume activated fibroblast (myofibroblast) characteristics [79]. In addition, in areas of collagenous deposition > 20 μm, the subepithelial band of macrophages is fragmented and gradually disappears [81].

At the molecular level, the CC mucosa secretes pro‐fibrotic basic fibroblast growth factor (bFGF) and vascular endothelial growth factor (VEGF). Since these can originate from IECs, inflammatory cells and fibroblasts in the lamina propria, myofibroblast differentiation and collagen deposition could result as a secondary outcome of the immune response [82, 83]. Subepithelial areas of excessive collagen deposition display an increased level of connective tissue growth factor (CTGF), tenascin, collagen type VI, and α‐smooth muscle actin (α‐SMA) indicating extracellular matrix (ECM) remodelling by pericryptal myofibroblasts (Table 1, Figure 1) [81, 84, 85]. However, mucosal gene expression of collagen genes remains unchanged, indicating a lack of *de novo* collagen synthesis [13]. Instead, matrix metalloproteinase inhibitors *TIMP1* and *TIMP3* are overexpressed, which points to a reduced ECM degradation [13, 86].

Of note, matrix metalloproteinase (MMP)‐9 is involved in epithelial damage in UC and correlates with the severity of mucosal damage [87]. Although Lakatos et al. failed to find differences in CC, *MMP9* has been genetically associated with CC and we previously reported a tendence towards increased *MMP9* gene expression in active CC [13, 87, 88]. In parallel, low serum FGF19 levels inversely correlate with the severity of diarrhoea independently of inflammation and are comparable in LC, CC, CD and UC [89].

Altogether, CC is a pericryptal myofibroblastic disorder with failure of ECM degradation in subepithelial areas that results in collagen deposition. This derives from a moderate, restrained inflammation that also promotes water malapsorption, osmoregulatory failures, and crypt hyperproliferation without mucosal macroscopic modifications.

## 7 Serotonin Could Accelerate Intestinal Motility and Perpetuate Inflammation in Microscopic Colitis

Serotonin or 5‐hydroxytryptamine (5‐HT) is a monoamine neurotransmitter produced in the gut by enterochromaffin cells (EEC), a type of enteroendocrine cell. While serotonin plays a pivotal role in immune cell activation and generation/perpetuation of inflammation in the gut, it also affects intestinal sensory‐motor and secretory functions, including chloride secretion [90]. In LC, serotonin is increased in both ascending and descending colons, suggesting a role in accelerating colonic motility and visceral hypersensitivity via the enteric nervous system [91]. Not only serotonin, but also serotonin synthesis initiating enzyme tryptophan hydroxylase 1 (TPH1) and its metabolite 5‐hydroxyindoeoacetic acid (5‐HIAA) are increased in LC mucosa and urine, respectively, together with an increased number of enteroendocrine cells. These can be returned to normal by treatment with budesonide assisted by reduced tryptophan dietary intake and, interestingly, 5‐HIAA correlates with severity of disease symptoms [92]. Similarly, peptide YY, a satiety signal produced by EECs and reaching the vagus nerve to control food intake, is increased in LC [93]. While peptide YY increase could be secondary to serotonin secretion, peptide YY production could compensate the accelerated motility by stimulating water and electrolyte absorption as well as the ‘ileal brake’ (a mechanism that reduces food intake due to detection of undigested nutrients in the ileum) [91, 94].

In CC, only a case report indicates high serotonin levels [95]. However, increased faecal levels of the EEC markers chromogranin A/B and secretoneurin are found in CC, even higher than those in CD and UC [96]. Therefore, EECs could also contribute to perpetuation of inflammation and symptoms in CC.

## 8 The Microbiota as a Disease Trigger

Genetic association of CC with *HLA* genes and transcriptomic alterations of genes related to antigen presentation, lipopolysaccharide response and IFN signalling routes point to a role for Gram‐negative bacteria and viruses in CC pathogenesis [6, 13]. Actually, CC displays an abnormal epithelial translocation of bacteria that could be responsible of triggering CC inflammation [97]. Microbiota studies so far have identified no changes or a modest decrease in mean species (alpha) diversity in MC, with no differences between LC and CC [98]. At taxonomic levels, an enrichment in plant‐diet and chronic inflammation associated with Prevotella genus (Gram‐negative), and decreased concentration of the epithelial‐protective *Akkermansia muciniphila* bacteria (Gram‐negative) and the Clostridia‐related butyrate‐producing Ruminococcaceae bacteria family (Gram‐positive) have been described for MC (Figure 1) [98, 99, 100, 101]. Especially, a decreased abundance of Ruminococcaceae is generally associated with loose stools and is restored after treatment with budesonide; hence, the bacterial microbiota might be affected by the luminal content flow [99, 102]. This profile is shared with other IBD forms [99, 102], but whether dysbiosis is causative or consequential to the inflammation remains unknown. Clearly, there is an association with microbiota—at least with CC –, as colonic lavage contributes to achieve remission after colonoscopic exploration, and faecal stream diversion has been effective in treating budesonide—nonresponsive CC patients [103, 104, 105].

In LC, there is an increased abundance of betaproteobacteria *Sutterella* (Gram‐negative), and a lower abundance of Clostridia‐related *Romboutsia* bacteria family (anaerobic Gram‐positive lipid‐producing bacteria) [102]. Interestingly, members of the genus *Sutterella* are widely prevalent commensals. Of note, *Sutterella* is capable of mild pro‐inflammatory responses—such as IL‐8 production by enterocytes and TNF‐α production by monocytes –, but do not contribute to dysbiosis‐associated epithelial dysfunction, loss of epithelial barrier integrity, or to IBD [106, 107]. Instead, *Sutterella* could motivate an alerted immune system in the host at an appropriate, physiological level [107]. Indeed, our transcriptomic analyses point towards a mild immune response in LC, lower than the response displayed in CC, which could match with this assumption [16]. Still, whether *Suterella* alone is enough to induce the lymphocytosis observed in LC remains unknown. Interestingly, infections with *Clostridium difficile*, *Escherichia* species and norovirus have been associated with a higher risk of LC (and to a lesser extent than CC) [108].

Epstein–Barr virus is nearly always detectable in MC biopsies, so it could explain the increased viral immune response found in LC and CC transcriptomes, and link both disease entities with autoimmunity (Figure 1) [13, 109]. In fact, it has been shown that the Epstein–Barr virus can trigger autoimmune responses by mimicry of host molecules, by infection of epithelial cells—including those in the gut—, and by modification of pivotal phenotypic of Th17 and IL‐17‐producing cells through signal re‐modelling in infected B‐cells [110, 111]. This pivotal Th17 activity could explain the increased Th17 cytokine overexpression in MC mucosa and apparent unchanged Th17 cell numbers [28, 29, 30]. In addition, ageing, vitamin D deficiency (common in autoimmune disorders and ageing), and high oestrogen levels (e.g. during pregnancy) reduce CD8^+^ T‐cell numbers and could contribute to trigger and/or maintain an immune response, and facilitate a lymphocytic infiltration in the affected organ [111, 112, 113]. Actually, this hypothesis could explain why MC is more frequent in elderly females and the association with other lymphocytic disorders of the gastrointestinal tract and autoimmune comorbidities [37, 114].

## 9 Future Perspectives

Diarrhoea is the main symptom for patients attending the gastroenterology consult. However, reaching a diagnosis for chronic diarrhoea—which has a prevalence of up to 5% in westernized populations—is challenging and can take years [115]. Of those, MC accounts for up to 15% of patients [116]. Still poorly understood, MC can be described as a non‐destructive, attenuated IBD. Basic research has been key to understanding MC pathomechanisms and identifying fundamental differences in genetic, immunological, histological, diarrhoeal, and microbial features between LC and CC [5]. Moreover, we recently proposed a subtype of LC lacking inflammation and purely based on ion channel and organic acid transport dysregulation (channelopathic LC), and a subtype with channelopathic and inflammatory alterations (inflammatory LC, Figure 1 and Table 1). In comparison, CC displays more proinflammatory features than LC despite the higher lymphocyte infiltration typical of LC mucosa [16]. Although these are interesting, yet provocative proposals, validation using different and larger patient cohorts will be indispensable to confirm immunopathogenic mechanisms in MC as well as distinctions between CC and LC.

MC mucosa remains macroscopically intact compared to classic IBD (CD and UC); hence, MC seems to restrain an overt immune response. Only in those patients with immune hyperactivation could MC progress towards an overt immune response that could damage the tissue and impair wound healing, and turn into classic IBD [45]. Therefore, MC could be taken as a unique proxy to study early stages of intestinal inflammation in IBD.

In the last decade, the rapid technological development has furthered high‐throughput sequencing and mass spectrometry. This has coined the term ‘multiomics’ to refer to the combination of techniques and methods to characterise cell states and activities by simultaneous integration of various profiling layers including (epi)genomics, (epi)transcriptomics, proteomics, metagenomics, metabolomics, immunomics or interactomics [117]. Emerging omics currently aim at exploring samples at single‐cell and/or spatial resolution coupling existing omics with high resolution imaging (i.e. spatial transcriptomics, spatial metabolomics, spatial metagenomics) [118, 119, 120]. To exploit such amounts of data, the research community is developing interactive tools such as Gut Cell Atlas (gutcellatlas.org) to make data available and more accessible [121]. These ‘top‐down’ approaches dissect complex disorders in spatiotemporal dynamic multilayer deconvolutions that uncover intricate molecular mechanisms underlying different phenotypic manifestations [117]. In IBD, multiomic integration, bioinformatics and artificial intelligence‐based system biology are approaching precision medicine to break the current therapeutic ceiling. This will allow patient stratification according to specific pathomechanisms to identify the best therapeutical, customised approach and prediction of treatment response [122]. Since MC is underexplored, research options are vast to advance the knowledge of pathomechanisms and identify new druggable targets.

A biobank is a biorepository that accepts, processes, stores, and distributes biospecimens and associated data for use in research and clinical care. From university‐based repositories that cover specific projects, there has been a gradual evolution towards institutional and government supported repositories, commercial biorepositories, population based biobanks, and virtual biobanks [123, 124]. These modern biobanks ensure safety, reliability, efficiency and trust by fulfiling ISO standardised procedures [125]. Under the umbrella of the European Microscopic Colitis Group (EMCG), we are developing a biobank network from already existing country‐specific MC or IBD biobanks. This will allow us to increase the sample size for basic and translational research using preserved samples that were collected and stored under standardised and validated procedures.

## 10 Conclusions

Microscopic colitis is comprised of two distinct disease entities (collagenous and lymphocytic colitis) whose clinical presentation is undiscernible and associated with IBD and autoimmunity. However, genetic, transcriptomic, histological and immunological studies clearly differentiate CC from LC. CC is associated with *HLA* gene locus and displays a mixed Th1/Tc1–Th17/Tc17 profile with the involvement of *AQP8*, *SLC9A3*/NHE3 and *SLC26A3*/DRA as drivers of water malabsorption and secondary fluid loss. In addition, subepithelial collagen deposition—a unique hallmark of CC—, results from myofibroblast activity in pericryptal areas and because of decreased extracellular matrix degradation. On the other hand, LC lacks genetic associations and displays a Th1/Th2 profile with both paracellular and transcellular ion transport dysregulation. Moreover, LC can be subclassified into channelopathic LC due to the unique alteration of ion and organic acid transport that could result from exposure to different drugs, and inflammatory LC due to the additional involvement of an immune response. However, the LC immune response is more moderate than that of CC despite its larger lymphocytic infiltrate. As MC mucosa remains intact and immune cells seem to stay inactive, MC is an ideal model to explore early stages of intestinal inflammation in IBD, provided that CC and LC are studied as distinct disease entities.

With the support of recently developed multiomic approaches and established biobanks, validation of MC patient stratification and further deepening into immunopathophysiological mechanisms and disease progression will eventually help to apply precision medicine to MC and IBD.

## Conflicts of Interest

A.M. has received salary for consultancies from Tillotts Pharma AG, Ferring, Vifor and Dr Falk Pharma; speaker’s honoraria from Tillotts Pharma AG and Vifor. The remaining authors declare no conflicts of interest.

## Data Availability

Data sharing is not applicable to this article as no new data were created or analysed in this study.
